# Endometriosis-Associated Implantation Failure: Pathophysiology, Biomarkers, and Emerging Therapeutic Strategies

**DOI:** 10.7759/cureus.113299

**Published:** 2026-07-24

**Authors:** Vaia Sarli, Charikleia Papageorgiou, Chrysi Christodoulaki, Periklis Panagopoulos, Nikolaos Machairiotis

**Affiliations:** 1 Third Department of Obstetrics and Gynecology, Attikon University General Hospital, Athens, GRC; 2 Department of Obstetrics and Gynecology, General Hospital of Chania, Chania, GRC; 3 Third Department of Obstetrics and Gynecology, National and Kapodistrian University of Athens Medical School, Attikon University General Hospital, Athens, GRC

**Keywords:** bcl6, endometrial receptivity, endometriosis, implantation failure, uterine natural killer cells

## Abstract

Implantation failure remains a major challenge in assisted reproductive technology (ART) and is a significant contributor to infertility in women with endometriosis. Beyond mechanical distortion and ovarian dysfunction, increasing evidence suggests that endometriosis profoundly alters endometrial receptivity through complex inflammatory, immunological, and molecular mechanisms. Chronic inflammation, progesterone resistance, dysregulated immune cell populations, and aberrant cytokine signaling converge to create a hostile endometrial microenvironment that compromises embryo-endometrial crosstalk and implantation success.

This narrative review provides a comprehensive overview of the pathophysiological pathways linking endometriosis to implantation failure, with particular emphasis on immune dysregulation and defects in endometrial receptivity. We summarize the current evidence regarding key immune cell populations, including uterine natural killer (uNK) cells and macrophages, and their role in modulating implantation. We further discuss established and emerging biomarkers of impaired receptivity, including BCL6, integrins, endometrial receptivity assays, and epigenetic markers, highlighting their diagnostic potential and current limitations.

Finally, we review contemporary and emerging therapeutic strategies aimed at restoring endometrial receptivity in women with endometriosis-associated implantation failure. These include hormonal pretreatment protocols, immunomodulatory approaches, and novel regenerative therapies such as platelet-rich plasma (PRP) and granulocyte colony-stimulating factor (G-CSF). By integrating mechanistic insights with clinical evidence, this review aims to provide a practical framework for the personalized management of implantation failure in women with endometriosis and to identify key directions for future research in this rapidly evolving field.

## Introduction and background

Endometriosis is a chronic inflammatory disease affecting approximately 10% of women of reproductive age and represents one of the leading causes of female infertility. Beyond its well-established association with pelvic pain and anatomical distortion, endometriosis has increasingly been recognized as a systemic and endometrial disorder that profoundly interferes with reproductive function. Women with endometriosis exhibit reduced spontaneous conception rates and significantly lower success rates following assisted reproductive technology (ART), even in the absence of severe pelvic adhesions or ovarian compromise [1].

Implantation failure remains a major limiting factor in ART outcomes and constitutes a particularly challenging clinical entity in women with endometriosis. Traditionally, impaired fertility in endometriosis has been attributed to mechanical factors, altered folliculogenesis, and compromised oocyte quality. However, accumulating evidence indicates that defective endometrial receptivity plays a central role in implantation failure in this population. Alterations in hormonal responsiveness, chronic inflammation, immune dysregulation, and aberrant molecular signaling converge to create a hostile endometrial microenvironment that impairs embryo-endometrium crosstalk and compromises implantation.

Endometrial receptivity is a finely regulated process that depends on coordinated endocrine, immune, and molecular events occurring within a narrow window of implantation. In women with endometriosis, progesterone resistance, altered expression of receptivity markers, dysregulated immune cell populations, and persistent inflammatory activation have been consistently reported. These abnormalities not only disrupt decidualization and trophoblast invasion but may also lead to repeated implantation failure (RIF), even when high-quality embryos are transferred. A range of molecular and immune biomarkers have been proposed to identify impaired endometrial receptivity in this population. Nevertheless, their clinical utility remains controversial, and standardized diagnostic algorithms are still lacking [2].

Therapeutic strategies aiming to restore endometrial receptivity in women with endometriosis-associated implantation failure have evolved substantially over the past decade. Hormonal pretreatment protocols, immunomodulatory interventions, and novel regenerative approaches such as platelet-rich plasma (PRP) and granulocyte colony-stimulating factor (G-CSF) have been increasingly explored, with heterogeneous and often conflicting results. The absence of consensus regarding optimal patient selection and treatment sequencing continues to represent a major unmet need in clinical practice.

This narrative review aims to provide a comprehensive overview of the pathophysiological mechanisms linking endometriosis to implantation failure, with particular emphasis on immune dysregulation and endometrial receptivity defects. We further summarize current evidence regarding established and emerging biomarkers of impaired receptivity and critically discuss available and novel therapeutic strategies. By integrating mechanistic insights with clinical data, this review seeks to propose a practical framework for the personalized management of implantation failure in women with endometriosis.

## Review

Endometriosis and impaired endometrial receptivity: progesterone resistance and molecular alterations

Progesterone signaling is a central determinant of endometrial receptivity and successful implantation, orchestrating decidualization, immune tolerance, and trophoblast invasion. In women with endometriosis, mounting evidence indicates the presence of progesterone resistance, characterized by impaired progesterone receptor signaling and downstream transcriptional dysregulation. This phenomenon has emerged as a key pathophysiological mechanism underlying defective endometrial receptivity and implantation failure in this population [1].

At the molecular level, altered expression and an imbalance in the progesterone receptors (PR-A and PR-B) have been consistently demonstrated in the eutopic endometrium of women with endometriosis. A relative predominance of PR-A over PR-B has been associated with attenuated progesterone responsiveness and defective decidualization. These alterations are further compounded by epigenetic modifications, including promoter hypermethylation and changes in histone acetylation, which impair progesterone-dependent gene transcription and contribute to persistent estrogenic dominance within the endometrial environment.

One of the most extensively studied downstream targets of progesterone signaling is the homeobox gene HOXA10, a critical regulator of endometrial differentiation and embryo implantation. Reduced HOXA10 expression has been repeatedly observed in the mid-luteal endometrium of women with endometriosis and correlates with impaired decidualization and decreased implantation potential. Similar dysregulation has been reported for HOXA11, further supporting the concept of a globally altered transcriptional program governing endometrial receptivity [3,4]. Integrins constitute another key group of molecular mediators affected in endometriosis-associated implantation failure. The alpha-v-beta-3 integrin, widely regarded as a canonical marker of endometrial receptivity, exhibits reduced or delayed expression during the window of implantation in women with endometriosis.

This aberrant integrin profile disrupts embryo adhesion and compromises early implantation events, independently of embryo quality [5]. Beyond receptor and adhesion molecule dysregulation, endometriosis is associated with profound alterations in the decidualization cascade. Decreased expression of prolactin, insulin-like growth factor binding protein-1, and forkhead box O1 reflects defective stromal cell differentiation and impaired acquisition of a receptive phenotype. These abnormalities are further exacerbated by persistent inflammatory activation and oxidative stress, which interfere with progesterone-mediated transcription and perpetuate a hostile endometrial microenvironment.

Inflammatory and oxidative stress pathways

Endometriosis is increasingly recognized as a chronic inflammatory condition characterized by sustained activation of immune and inflammatory pathways within both ectopic lesions and the eutopic endometrium. This persistent inflammatory state profoundly alters the endometrial microenvironment and represents a major determinant of impaired receptivity and implantation failure. At the cellular level, eutopic endometrium from women with endometriosis exhibits increased infiltration of activated macrophages, neutrophils, and mast cells, accompanied by excessive production of pro-inflammatory mediators.

Elevated concentrations of interleukins, tumor necrosis factor-α, and chemokines have been consistently detected during the peri-implantation period, disrupting the tightly regulated inflammatory balance required for successful embryo implantation. While physiological implantation requires a transient and finely tuned inflammatory response, the chronic inflammatory state observed in endometriosis leads to sustained activation that becomes detrimental to endometrial function [5].

Cyclooxygenase-2 (COX-2) overexpression represents a central molecular hallmark of endometriosis-associated inflammation. Increased COX-2 activity results in excessive prostaglandin synthesis, particularly prostaglandin E2, which promotes angiogenesis, vascular permeability, and leukocyte recruitment. In the context of implantation, prostaglandin dysregulation interferes with endometrial differentiation, alters vascular remodeling, and perturbs the molecular dialogue between the embryo and the endometrium. Experimental models have further demonstrated that aberrant prostaglandin signaling compromises trophoblast invasion and impairs early placentation. Oxidative stress constitutes an additional and closely interconnected pathogenic pathway. Increased generation of reactive oxygen species and impaired antioxidant defenses have been documented in both peritoneal fluid and the eutopic endometrium of women with endometriosis.

Excessive oxidative stress induces lipid peroxidation, protein oxidation, and DNA damage, thereby altering cellular signaling and transcriptional programs essential for receptivity. Importantly, oxidative stress directly interferes with progesterone signaling pathways and amplifies progesterone resistance, further aggravating molecular defects in decidualization and implantation. Beyond their direct cellular effects, inflammatory and oxidative pathways profoundly remodel the endometrial extracellular matrix and vascular architecture. Altered matrix metalloproteinase activity, aberrant angiogenesis, and endothelial dysfunction have been described in the receptive phase, leading to impaired stromal remodeling and suboptimal trophoblast anchoring. These microenvironmental alterations compromise embryo apposition and adhesion independently of embryo competence.

Immunological mechanisms linking endometriosis to implantation failure

Uterine natural killer (uNK) cells represent the predominant immune cell population within the endometrium during the peri-implantation period and play a pivotal role in regulating implantation, decidualization, and early placentation. Unlike peripheral cytotoxic NK cells, uNK cells exhibit a specialized phenotype characterized by reduced cytotoxicity and enhanced secretory capacity, contributing to vascular remodeling, immune tolerance, and trophoblast invasion. In women with endometriosis, both quantitative and functional alterations in uNK cells have been increasingly implicated in the pathogenesis of implantation failure [6].

Several studies have demonstrated aberrant uNK cell density and distribution within the eutopic endometrium of women with endometriosis, particularly during the mid-luteal phase. Increased numbers of uNK cells have been reported in a subset of patients with RIF, whereas other investigations have identified qualitative defects rather than numerical abnormalities. These discrepancies likely reflect methodological heterogeneity and highlight the importance of functional profiling over absolute cell counts.

At the functional level, uNK cells in endometriosis exhibit altered cytotoxic potential and impaired secretion of angiogenic and immunoregulatory mediators. Dysregulated expression of killer-cell immunoglobulin-like receptors and their cognate human leukocyte antigen ligands on trophoblast cells disrupts the finely tuned receptor-ligand interactions required for immune tolerance and vascular adaptation. Aberrant KIR-HLA combinations have been associated with defective spiral artery remodeling, shallow trophoblast invasion, and compromised placentation, thereby predisposing to implantation failure and early pregnancy loss [7,8]. In addition to receptor-mediated signaling, endometriosis-associated inflammation profoundly modulates uNK cell function.

Elevated local concentrations of pro-inflammatory cytokines, including interleukin-6 and tumor necrosis factor-α, shift uNK cells toward a more cytotoxic phenotype and attenuate their pro-angiogenic activity. This phenotypic reprogramming disrupts the balance between immune surveillance and immune tolerance that is essential for successful implantation. Emerging evidence further suggests that progesterone resistance contributes to uNK cell dysregulation in endometriosis. Progesterone-dependent induction of key immunomodulatory molecules, such as glycodelin and galectin-1, is attenuated in the receptive endometrium, thereby impairing uNK-mediated immune tolerance and trophoblast accommodation. These hormonal-immune interactions provide an additional mechanistic link between endocrine dysfunction and immune-mediated implantation failure.

Macrophages and antigen-presenting cells

Macrophages constitute a major immune cell population within the endometrial stroma and play a central role in tissue remodeling, angiogenesis, immune tolerance, and regulation of trophoblast invasion. During normal implantation, macrophages undergo dynamic polarization toward an anti-inflammatory and pro-reparative M2 phenotype, facilitating decidualization, extracellular matrix remodeling, and vascular adaptation. In women with endometriosis, profound quantitative and qualitative alterations in endometrial macrophage populations have been increasingly recognized as key contributors to implantation failure [9]. Endometriosis is characterized by increased recruitment and activation of macrophages within both ectopic lesions and eutopic endometrium.

Elevated macrophage density has been consistently reported during the peri-implantation period, reflecting sustained inflammatory activation and aberrant immune surveillance. Importantly, beyond numerical expansion, endometrial macrophages in endometriosis exhibit a marked polarization imbalance, with a predominance of pro-inflammatory M1 phenotypes and relative deficiency of immunoregulatory M2 subsets. This skewed polarization disrupts the physiological immune tolerance required for embryo implantation and promotes persistent tissue inflammation [10,11].

At the molecular level, M1-polarized macrophages secrete high concentrations of pro-inflammatory cytokines, reactive oxygen species, and matrix-degrading enzymes that impair stromal differentiation and compromise extracellular matrix integrity. Excessive production of tumor necrosis factor-α, interleukin-1β, and nitric oxide interferes with decidualization and inhibits trophoblast migration, thereby directly impairing early implantation events. In parallel, reduced M2 macrophage activity attenuates the secretion of angiogenic and growth-promoting factors, including vascular endothelial growth factor and transforming growth factor-β, which are essential for spiral artery remodeling and placental anchoring.

Macrophage-mediated fibrosis represents an additional pathogenic mechanism linking endometriosis to implantation failure. Activated macrophages promote fibroblast proliferation and collagen deposition through the release of profibrotic mediators, resulting in increased stromal stiffness and altered biomechanical properties of the endometrium. Such fibrotic remodeling compromises embryo apposition and invasion and has been associated with reduced implantation potential independently of hormonal and embryonic factors.

Antigen-presenting cells, including dendritic cells, further contribute to immune dysregulation in endometriosis. Under physiological conditions, uterine dendritic cells participate in antigen tolerance and promote regulatory T-cell expansion, thereby facilitating maternal-fetal immune adaptation. In endometriosis, aberrant maturation and activation of dendritic cells have been reported, leading to enhanced antigen presentation, impaired tolerogenic signaling, and defective induction of regulatory T cells.

This shift toward immunostimulatory phenotypes perpetuates chronic inflammation and undermines immune tolerance at the maternal-embryonic interface. Taken together, macrophage polarization imbalance and antigen-presenting cell dysfunction represent central immunopathological mechanisms underlying defective implantation in endometriosis. Their wide-ranging effects on inflammation, fibrosis, angiogenesis, and immune tolerance highlight their pivotal role in the pathogenesis of endometriosis-associated implantation failure and support their potential as diagnostic biomarkers and therapeutic targets.

Cytokine and chemokine networks

Successful implantation requires a finely regulated cytokine and chemokine milieu that orchestrates immune tolerance, stromal differentiation, angiogenesis, and trophoblast invasion. This tightly controlled network ensures a transient pro-inflammatory phase during embryo apposition, followed by a rapid shift toward an anti-inflammatory and immunotolerant environment that supports decidualization and placental development. In women with endometriosis, profound dysregulation of cytokine and chemokine signaling has emerged as a central mechanism underlying impaired receptivity and implantation failure.

Multiple studies have demonstrated an aberrant cytokine profile within the eutopic endometrium and peritoneal fluid of women with endometriosis, characterized by sustained elevation of pro-inflammatory mediators throughout the menstrual cycle. Increased concentrations of interleukin-1β, interleukin-6, tumor necrosis factor-α, and interferon-γ have been consistently reported during the peri-implantation period, reflecting persistent immune activation that disrupts the physiological temporal pattern required for implantation. These cytokines directly impair decidualization, inhibit trophoblast invasion, and alter endothelial function, thereby compromising multiple steps of early implantation.

The Th1/Th2 balance represents a critical determinant of implantation success. Under physiological conditions, a transient Th1-dominant inflammatory response facilitates embryo attachment, followed by a Th2-biased immunotolerant state that supports trophoblast invasion and placental development. In endometriosis, a persistent Th1-skewed immune profile has been observed, with excessive production of Th1 cytokines and relative suppression of Th2 and regulatory mediators. This sustained pro-inflammatory polarization impairs immune tolerance at the maternal-embryonic interface and predisposes to implantation failure and early pregnancy loss [12].

Chemokine signaling further contributes to immune dysregulation in endometriosis. Altered expression of chemokines such as CXCL12, CCL2, and CCL5 disrupts the spatial recruitment and positioning of immune cells within the endometrial stroma. Aberrant chemokine gradients impair the coordinated trafficking of uNK cells, macrophages, and regulatory T cells, thereby perturbing local immune architecture and compromising the establishment of a receptive microenvironment. Dysregulated chemokine signaling has additionally been implicated in defective angiogenesis and abnormal vascular patterning during the window of implantation [13].

Beyond classical inflammatory mediators, emerging evidence highlights the role of regulatory cytokines in modulating implantation competence. Reduced expression of interleukin-10 and transforming growth factor-β has been reported in the receptive endometrium of women with endometriosis, reflecting impaired induction of immune tolerance and defective expansion of regulatory T-cell populations. These deficiencies exacerbate local immune activation and undermine the establishment of maternal-fetal tolerance during the earliest stages of pregnancy [14]. Importantly, cytokine and chemokine networks interact closely with hormonal and metabolic signaling pathways. Progesterone resistance amplifies pro-inflammatory cytokine production and attenuates the anti-inflammatory effects of progesterone-dependent mediators, thereby reinforcing immune dysregulation.

In parallel, oxidative stress further activates nuclear factor-κB and other transcription factors that drive cytokine gene expression, sustaining chronic inflammatory activation throughout the implantation window. Collectively, dysregulated cytokine and chemokine networks represent a central integrative mechanism linking hormonal resistance, immune activation, and microenvironmental remodeling in endometriosis-associated implantation failure. Their diverse effects on immune cell recruitment, angiogenesis, stromal differentiation, and trophoblast invasion highlight their potential as both diagnostic biomarkers and therapeutic targets.

Biomarkers of endometrial dysfunction in endometriosis

Transcriptomic and Molecular Biomarkers

The identification of reliable biomarkers of impaired endometrial receptivity represents a major priority in the management of implantation failure associated with endometriosis. Advances in transcriptomic profiling and molecular diagnostics have revealed profound alterations in gene expression within the eutopic endometrium of affected women, reflecting the underlying hormonal resistance, inflammatory activation, and immune dysregulation that characterize this condition. Among the proposed molecular biomarkers, transcription factors and progesterone-responsive genes have emerged as particularly informative indicators of defective receptivity.

One of the most extensively investigated biomarkers in this context is B-cell lymphoma 6 (BCL6), a transcriptional repressor that has been increasingly implicated in progesterone resistance and endometrial dysfunction. BCL6 is overexpressed in the eutopic endometrium of women with endometriosis, particularly during the mid-luteal phase, and has been shown to inhibit progesterone receptor signaling and downstream decidualization pathways [15]. Mechanistically, BCL6 suppresses the expression of key progesterone-regulated genes, including HOXA10 and Indian hedgehog, thereby disrupting stromal differentiation and impairing the acquisition of a receptive phenotype.

Clinical studies have demonstrated a strong association between endometrial BCL6 overexpression and RIF, even in women without laparoscopically confirmed endometriosis. Elevated BCL6 expression has been proposed as a surrogate marker of occult endometriosis and inflammatory progesterone resistance, identifying a subset of patients with otherwise unexplained implantation failure who may benefit from targeted pretreatment strategies. Importantly, BCL6 expression has been shown to normalize following medical or surgical suppression of endometriosis, further supporting its role as a dynamic biomarker reflecting disease activity and treatment response [16,17].

Beyond BCL6, multiple progesterone-responsive genes involved in decidualization and implantation are dysregulated in endometriosis. Reduced expression of HOXA10 and HOXA11, leukemia inhibitory factor, and glycodelin has been consistently reported during the window of implantation, reflecting defective transcriptional programming of endometrial stromal cells. These alterations impair embryo adhesion, trophoblast invasion, and immune tolerance, thereby directly compromising implantation competence [3,4].

High-throughput transcriptomic analyses have further revealed global reprogramming of endometrial gene expression in women with endometriosis. Differential expression of genes involved in cell adhesion, angiogenesis, immune regulation, and extracellular matrix remodeling has been documented, demonstrating the multifactorial nature of receptivity defects. Notably, aberrant activation of inflammatory signaling pathways, including nuclear factor-κB and signal transducer and activator of transcription networks, has been identified as a recurrent molecular signature linking inflammation to progesterone resistance and implantation failure [18].

Despite their strong biological rationale, the clinical implementation of transcriptomic biomarkers remains challenging. Variability in sampling timing, menstrual cycle heterogeneity, and methodological differences in gene expression platforms contribute to inconsistent diagnostic performance across studies. Furthermore, the absence of standardized cut-off values and prospective validation limits the widespread adoption of these biomarkers in routine clinical practice. Among them, BCL6 has emerged as a particularly robust indicator of inflammatory progesterone resistance and represents a potential cornerstone biomarker in the diagnostic algorithm for implantation failure in women with endometriosis [15,16].

Endometrial Receptivity Testing

The concept of a personalized window of implantation has led to the development of endometrial receptivity assays aimed at identifying temporal displacement or molecular dysfunction of the receptive phase in women with implantation failure. Among these, transcriptomic-based endometrial receptivity testing has gained considerable attention as a potential tool for optimizing embryo transfer timing and improving ART outcomes. In the context of endometriosis, however, the diagnostic performance and clinical utility of such assays remain controversial [19].

The endometrial receptivity array (ERA) represents the most extensively studied receptivity assay and evaluates the expression profile of a predefined panel of genes associated with the receptive endometrial phenotype. By classifying endometrial samples as receptive or non-receptive, ERA aims to identify a personalized window of implantation and guide individualized embryo transfer timing. Several studies have reported a higher prevalence of displaced windows of implantation in women with RIF and in those with endometriosis, suggesting that temporal asynchrony may contribute to implantation failure in this population [20].

In women with endometriosis, transcriptomic analyses have consistently demonstrated altered expression of receptivity-related genes during the mid-luteal phase, reflecting underlying progesterone resistance and inflammatory activation. These molecular perturbations raise the possibility that conventional timing based on hormonal exposure may not adequately capture the receptive phase in affected patients. Accordingly, a subset of studies has reported improved implantation and pregnancy rates following personalized embryo transfer guided by receptivity testing in women with endometriosis and RIF.

Nevertheless, substantial limitations undermine the routine clinical application of receptivity assays in this setting. Inter-cycle variability in gene expression, hormonal fluctuations, and inflammatory activity may lead to inconsistent receptivity profiles, particularly in women with chronic inflammatory conditions such as endometriosis. Furthermore, the transcriptomic signature of receptivity in endometriosis may reflect a qualitative defect rather than a purely temporal displacement, thereby limiting the corrective potential of timing adjustments alone [21,22].

Importantly, receptivity assays do not capture the full spectrum of pathophysiological alterations associated with endometriosis. Immune dysregulation, cytokine imbalance, and microenvironmental remodeling, which play central roles in implantation failure, are only partially reflected in current transcriptomic panels. Evidence from randomized controlled trials and meta-analyses has failed to demonstrate a consistent benefit of receptivity-guided embryo transfer in unselected ART populations, and available data in women with endometriosis remain limited by small sample sizes and lack of standardized protocols [22,23]. Integration of receptivity testing with immune profiling and biomarker-guided pretreatment strategies may represent a more comprehensive approach for patient stratification and personalized management.

MicroRNAs and Epigenetic Regulation

Epigenetic regulation has emerged as a fundamental mechanism governing endometrial receptivity, immune tolerance, and decidualization. Increasing attention has focused on the role of microRNAs (miRNAs) and epigenetic modifications in mediating the molecular alterations associated with endometriosis and implantation failure. These regulatory layers provide a mechanistic link between chronic inflammation, progesterone resistance, and persistent transcriptional reprogramming of the endometrium [23]. MicroRNAs are small non-coding RNAs that post-transcriptionally regulate gene expression by targeting messenger RNA stability and translation. In the receptive endometrium, tightly coordinated miRNA expression patterns modulate key pathways involved in cell adhesion, angiogenesis, immune regulation, and hormonal responsiveness. In women with endometriosis, aberrant miRNA profiles have been consistently identified within eutopic endometrium, reflecting disease-specific epigenetic reprogramming that compromises implantation competence.

Among the most extensively studied miRNAs, miR-135a has been shown to directly suppress HOXA10 expression, thereby impairing progesterone-dependent transcriptional programming and decidualization. Overexpression of miR-135a in the mid-luteal endometrium of women with endometriosis correlates with reduced HOXA10 levels and decreased implantation potential, highlighting a direct epigenetic mechanism linking endometriosis to defective receptivity. Similarly, dysregulation of miR-451 and miR-29 family members has been implicated in altered inflammatory signaling and extracellular matrix remodeling, further compromising stromal differentiation and embryo adhesion [24].

Beyond individual miRNAs, global alterations in epigenetic landscapes have been documented in endometriosis-associated endometrial dysfunction. Aberrant DNA methylation patterns affecting progesterone receptor promoters, HOXA gene clusters, and immune-regulatory loci contribute to sustained progesterone resistance and persistent inflammatory activation. Histone modifications, including altered acetylation and methylation states, further modulate chromatin accessibility and transcription factor binding, reinforcing pathological gene expression programs during the window of implantation [25].

Importantly, epigenetic dysregulation in endometriosis appears to be dynamic and potentially reversible. Medical and surgical suppression of disease activity has been shown to partially restore normal methylation patterns and miRNA expression profiles, suggesting that epigenetic biomarkers may reflect both disease burden and treatment response. This plasticity confers particular clinical relevance to epigenetic profiling as a tool for patient stratification and monitoring of therapeutic efficacy. From a translational perspective, miRNAs and epigenetic signatures offer several advantages as biomarkers of implantation failure. Their relative stability, detectability in endometrial tissue and uterine fluid, and close association with key pathogenic pathways render them attractive candidates for non-invasive diagnostics and personalized treatment selection. However, significant challenges remain, including inter-cycle variability, technical heterogeneity, and the lack of standardized analytical platforms [26].

Immune and Inflammatory Biomarkers

Immune and inflammatory biomarkers have emerged as promising tools for the identification of endometrial dysfunction in women with endometriosis-associated implantation failure. Given the central role of immune dysregulation and chronic inflammation in the pathogenesis of defective receptivity, profiling of immune cell populations and inflammatory mediators has been increasingly explored as a means of refining diagnosis, stratifying patients, and guiding personalized therapeutic interventions. Assessment of uNK cell density and activity represents one of the most extensively investigated immune biomarkers in implantation failure.

Increased uNK cell numbers, altered phenotypic profiles, and enhanced cytotoxic activity have been reported in subsets of women with RIF and endometriosis. Immunohistochemical quantification of CD56-positive cells and flow cytometric analysis of NK receptor expression have been proposed as diagnostic tools; however, substantial methodological variability and lack of standardized reference ranges limit their clinical applicability. Moreover, uNK cell number alone fails to capture functional competence, and discordance between cell density and cytotoxic potential has been frequently observed [6,7].

Cytokine profiling constitutes another widely studied approach to immune biomarker development. Elevated endometrial and uterine fluid concentrations of pro-inflammatory cytokines, including interleukin-6, interleukin-1β, tumor necrosis factor-α, and interferon-γ, have been consistently associated with implantation failure and adverse reproductive outcomes. Conversely, reduced levels of regulatory mediators such as interleukin-10 and transforming growth factor-β reflect impaired immune tolerance and defective expansion of regulatory T-cell populations [13,14]. Recent advances in immune profiling technologies have enabled more comprehensive characterization of endometrial immune landscapes. Multiparametric flow cytometry, single-cell RNA sequencing, and spatial transcriptomics have revealed complex immune cell heterogeneity within the receptive endometrium and identified disease-specific immune signatures in endometriosis.

Altered proportions of regulatory T cells, dysfunctional macrophage subsets, and aberrant dendritic cell maturation profiles have been correlated with implantation failure and disease severity. These high-dimensional approaches provide unprecedented resolution but remain largely confined to research settings due to cost, technical complexity, and limited standardization. Importantly, immune biomarkers are inherently dynamic and influenced by hormonal milieu, inflammatory activity, and therapeutic interventions. The absence of validated cut-off values and prospective outcome-driven studies precludes routine clinical implementation. Integration of immune profiling with molecular and transcriptomic biomarkers may enable the identification of biologically distinct endotypes of implantation failure, facilitating tailored therapeutic strategies.

Therapeutic strategies for endometriosis-associated implantation failure

Hormonal Pretreatment Approaches

Hormonal pretreatment represents one of the most extensively investigated strategies for improving implantation outcomes in women with endometriosis-associated implantation failure. The rationale for hormonal suppression is grounded in the pathophysiology of the disease, aiming to attenuate inflammatory activity, reverse progesterone resistance, and restore endometrial receptivity before embryo transfer. Among available approaches, gonadotropin-releasing hormone (GnRH) agonist-based protocols have received the greatest attention.

Prolonged pituitary suppression with GnRH agonists, commonly referred to as ultra-long protocols, has been proposed as a means of reducing ectopic lesion activity, suppressing inflammatory mediators, and normalizing endometrial gene expression. Several observational studies and randomized trials have reported improved implantation, clinical pregnancy, and live birth rates in women with moderate to severe endometriosis undergoing in vitro fertilization following two to six months of GnRH agonist pretreatment. These benefits appear to be particularly pronounced in patients with advanced disease and in those with a history of RIF [27].

At the molecular level, GnRH agonist suppression has been shown to downregulate inflammatory cytokines, reduce prostaglandin synthesis, and partially restore progesterone responsiveness within the eutopic endometrium. Normalization of key receptivity markers, including HOXA10 and integrins, has been documented following prolonged suppression, supporting a mechanistic basis for improved implantation competence. Furthermore, medical suppression has been associated with reduced BCL6 expression, suggesting reversal of inflammatory progesterone resistance in selected patients.

Despite these promising findings, the efficacy of GnRH agonist pretreatment remains heterogeneous and patient-dependent. Meta-analyses have demonstrated a modest but significant improvement in clinical pregnancy rates; however, substantial variability in study design, disease stage, pretreatment duration, and ART protocols limits the generalizability of results. The benefit of ultra-long suppression appears less consistent in women with minimal or mild endometriosis and in those without overt inflammatory activity [28].

Alternative hormonal strategies, including oral progestins, combined oral contraceptives, and dienogest-based suppression, have been explored with variable success. Progestin pretreatment may exert anti-inflammatory and anti-proliferative effects on ectopic lesions and improve luteal-phase endometrial differentiation; however, evidence regarding implantation outcomes remains limited and largely derived from small observational cohorts. Dienogest, in particular, has shown efficacy in reducing pain and lesion burden, but its impact on endometrial receptivity and ART success remains incompletely defined [29].

Importantly, hormonal pretreatment is not devoid of limitations. Prolonged suppression may adversely affect ovarian reserve, delay treatment, and increase patient burden. Hypoestrogenic side effects and impaired endometrial recovery following extended suppression may further compromise implantation if inadequate washout intervals are applied. These considerations emphasize the need for careful patient selection and individualized protocol design [30].

Immunomodulatory Therapies

Given the central role of immune dysregulation in the pathogenesis of endometriosis-associated implantation failure, immunomodulatory therapies have been widely explored as adjunctive strategies in assisted reproduction. These interventions aim to attenuate excessive inflammatory activation, restore immune tolerance at the maternal-embryonic interface, and modulate aberrant immune cell function. However, despite their widespread clinical use, robust evidence supporting their efficacy remains limited and controversial.

Corticosteroids represent the most commonly employed immunomodulatory agents in reproductive medicine. By suppressing pro-inflammatory cytokine production and inhibiting lymphocyte activation, corticosteroids are theoretically expected to improve implantation by restoring immune balance and reducing endometrial inflammation [31]. Several small observational studies have reported improved implantation and pregnancy rates in women with RIF and elevated immune activation markers.

Nevertheless, randomized controlled trials and meta-analyses have failed to demonstrate consistent benefit in unselected ART populations. Intralipid therapy has been proposed as a means of modulating uNK cell activity and reducing cytotoxic immune responses. Experimental data suggest that intralipids may downregulate NK cell cytotoxicity and alter cytokine secretion profiles. However, evidence supporting its use remains largely derived from uncontrolled studies and retrospective analyses, with conflicting results regarding implantation and live birth rates [32]. Reliable biomarkers for patient selection and treatment monitoring are lacking, limiting the rational application of this intervention.

Intravenous immunoglobulin (IVIG) has been investigated as a more potent immunomodulatory strategy in selected patients with severe immune dysregulation. IVIG exerts multifaceted effects on both innate and adaptive immunity, including modulation of NK cell activity, suppression of autoantibody production, and enhancement of regulatory T-cell function. Several early studies suggested improved pregnancy outcomes in women with RIF and abnormal immune profiles; however, subsequent trials yielded inconsistent results. The high cost, limited availability, and potential for adverse reactions further restrict the routine use of IVIG in clinical practice.

A major limitation of immunomodulatory therapies lies in the lack of standardized diagnostic criteria for immune-mediated implantation failure. Immune biomarkers exhibit substantial inter-cycle variability, and consensus regarding clinically relevant thresholds is lacking. Consequently, immunomodulation is frequently applied empirically, without clear mechanistic justification or evidence-based patient selection. Carefully designed trials incorporating immune phenotyping, biomarker-guided patient selection, and standardized outcome measures are urgently needed to define the role of targeted immunomodulation in personalized reproductive medicine [31,32].

Regenerative and Growth Factor-Based Therapies

The recognition of endometrial dysfunction as a central mechanism of implantation failure has stimulated growing interest in regenerative and growth factor-based therapies aimed at restoring receptivity and microenvironmental integrity. Among these approaches, PRP and G-CSF have emerged as the most extensively investigated regenerative interventions in women with RIF and endometrial insufficiency, including those with endometriosis. PRP represents an autologous concentration of platelets suspended in plasma and enriched with a broad spectrum of bioactive mediators, including platelet-derived growth factor, transforming growth factor-β, vascular endothelial growth factor, epidermal growth factor, and insulin-like growth factor. These factors exert wide-ranging effects on angiogenesis, stromal proliferation, extracellular matrix remodeling, and immune modulation, thereby recapitulating key pathways involved in physiological endometrial regeneration and receptivity [33].

At the mechanistic level, PRP has been shown to enhance endometrial stromal cell proliferation, promote decidualization, and upregulate the expression of receptivity markers, including HOXA10, leukemia inhibitory factor, and integrins. In parallel, PRP exerts potent immunomodulatory effects by attenuating pro-inflammatory cytokine production, promoting macrophage polarization toward anti-inflammatory phenotypes, and enhancing regulatory T-cell activity. These combined regenerative and immunoregulatory properties render PRP particularly attractive for the treatment of endometriosis-associated implantation failure, in which inflammation, progesterone resistance, and immune dysregulation coexist.

Clinical studies evaluating intrauterine PRP administration in women with RIF have reported encouraging improvements in endometrial thickness, implantation rates, and clinical pregnancy outcomes. Several prospective and retrospective cohorts have demonstrated higher implantation and live birth rates following PRP infusion in patients with thin endometrium or RIF, including subsets with suspected or confirmed endometriosis [34,35]. Despite these promising findings, available evidence remains limited by methodological heterogeneity, small sample sizes, and variable PRP preparation protocols. Well-designed randomized controlled trials focusing specifically on endometriosis-associated implantation failure are currently scarce [36].

G-CSF represents an alternative growth factor-based strategy with both hematopoietic and immunomodulatory properties. Beyond its classical role in neutrophil proliferation, G-CSF exerts direct effects on endometrial angiogenesis, stromal proliferation, and immune tolerance. Clinically, intrauterine and systemic G-CSF administration has been primarily investigated in women with thin endometrium and RIF. Several studies have reported increased endometrial thickness and improved implantation rates following G-CSF treatment, whereas others have failed to demonstrate significant benefit.

In women with endometriosis, evidence remains extremely limited, and the specific contribution of G-CSF to reversing inflammatory and epigenetic alterations of the endometrium has not been adequately explored [37,38]. Importantly, regenerative therapies may exert maximal benefit in biologically selected patient populations. Women exhibiting inflammatory progesterone resistance, elevated BCL6 expression, immune activation, or refractory endometrial thinning may represent optimal candidates for PRP or G-CSF intervention. Integration of regenerative therapies with biomarker-guided patient stratification holds particular promise for enhancing therapeutic efficacy and minimizing unnecessary treatment exposure.

Endometrial Scratching and Mechanical Interventions

Mechanical endometrial injury, commonly referred to as endometrial scratching, has been proposed as an adjunctive intervention aimed at enhancing implantation through induction of a localized inflammatory response and modulation of endometrial receptivity. Initial observational studies and small randomized trials suggested improved implantation and pregnancy rates in women with RIF. However, subsequent large randomized controlled trials and meta-analyses failed to confirm a consistent benefit in unselected ART populations, leading to a substantial reevaluation of its clinical utility [39].

In the context of endometriosis, the role of endometrial scratching remains particularly controversial. Given the chronic inflammatory state already present within the eutopic endometrium, additional mechanical injury may exacerbate immune activation and oxidative stress rather than restore receptivity. Available studies specifically addressing scratching in women with endometriosis are scarce and heterogeneous, and robust evidence supporting routine application in this population is lacking [40]. Current evidence does not support its routine use in this setting.

Clinical implications and management framework

The complex and multifactorial nature of endometriosis-associated implantation failure necessitates an integrated and personalized approach to diagnosis and treatment. A stepwise diagnostic framework should begin with careful phenotyping of patients presenting with RIF and suspected or confirmed endometriosis. In addition to conventional clinical and imaging assessment, evaluation of endometrial function through molecular and immune biomarkers - including progesterone resistance markers, BCL6 expression, inflammatory activity, and immune profiles - may facilitate identification of biologically distinct endotypes of implantation failure [41].

Pretreatment strategies should be guided by disease severity, inflammatory burden, and biomarker profiles. Adjunctive therapies such as PRP or G-CSF should be considered selectively and within a biomarker-guided framework. Routine use of empiric immunomodulatory therapies in unselected patients should be discouraged in the absence of clear immune-mediated pathology. Endometrial receptivity testing and immune profiling should be interpreted cautiously and integrated with clinical context rather than applied as isolated diagnostic tools.

Table 1 presents a proposed stepwise management framework based on patient phenotype, informed by current evidence. Prospective validation is required before formal guideline adoption [41,42,43].

**Table 1:** Proposed stepwise management framework for endometriosis-associated implantation failure ERA: endometrial receptivity array; FET: frozen embryo transfer; G-CSF: granulocyte colony-stimulating factor; GnRH: gonadotropin-releasing hormone; PRP: platelet-rich plasma; RIF: repeated implantation failure; uNK: uterine natural killer

Patient phenotype	Recommended pretreatment	Adjunctive therapy
Advanced endometriosis (stage III-IV) or pronounced inflammatory phenotype	GnRH agonist ultra-long protocol (2-6 months)	Consider PRP if endometrial thinning or refractory dysfunction present
Elevated BCL6 or inflammatory progesterone resistance	GnRH agonist suppression; target BCL6 normalization before transfer	Biomarker-guided reassessment; repeat endometrial sampling if needed
Minimal/mild endometriosis (stage I-II), no overt inflammation	Standard FET preparation; shorten or omit suppression phase	ERA testing if ≥2 prior unexplained failures
RIF with confirmed immune activation (elevated uNK, cytokine profile)	Hormonal suppression; consider targeted immunomodulation	Intralipid or corticosteroids only if immune biomarker criteria met
Thin endometrium (≤7 mm) refractory to standard preparation	Optimized oestrogen support protocol; extended preparation	Intrauterine G-CSF or PRP infusion

Limitations of current evidence and future perspectives

Despite substantial advances in understanding the pathophysiology of endometriosis-associated implantation failure, important limitations constrain the translation of current evidence into routine clinical practice. Most available studies are characterized by small sample sizes, heterogeneous patient populations, variable diagnostic criteria, and inconsistent outcome definitions, limiting reproducibility and generalizability. The lack of standardized biomarkers and validated thresholds represents a major barrier to precision medicine. Inter-cycle variability, technical heterogeneity, and incomplete understanding of temporal dynamics compromise the reliability of single-time-point measurements. Furthermore, the majority of therapeutic trials lack biomarker-guided patient selection, precluding identification of responders and obscuring true treatment effects.

Future research should prioritize prospective, multicenter trials incorporating rigorous phenotyping, standardized biomarker panels, and mechanistic endpoints. Integration of multi-omics approaches, including transcriptomics, epigenomics, and immune profiling, holds particular promise for defining biologically distinct endotypes of implantation failure and guiding targeted intervention. Emerging technologies such as single-cell sequencing, spatial transcriptomics, and minimally invasive diagnostic platforms, including uterine fluid biomarkers and peripheral immune signatures, may facilitate broader clinical implementation. Ultimately, the transition from empiric treatment paradigms to biomarker-driven, personalized reproductive care represents a central objective for improving outcomes in women with endometriosis-associated implantation failure.

## Conclusions

Endometriosis-associated implantation failure represents a complex clinical entity driven by the interaction of hormonal resistance, chronic inflammation, immune dysregulation, and persistent molecular reprogramming of the endometrium. Beyond mechanical distortion and oocyte impairment, defective endometrial receptivity emerges as a central determinant of reproductive failure in this population. Advances in reproductive immunology and molecular diagnostics have provided critical insights into the mechanisms linking endometriosis to impaired implantation. Alterations in progesterone signaling, immune cell function, cytokine networks, and epigenetic regulation collectively shape a hostile endometrial microenvironment that compromises embryo-endometrium crosstalk and placentation. While emerging biomarkers and regenerative therapies offer promising avenues for personalized intervention, current evidence emphasizes the need for cautious interpretation and selective clinical application. Integration of molecular, immune, and clinical parameters into individualized treatment algorithms represents the most rational strategy for optimizing implantation outcomes. Future progress will depend on rigorous mechanistic studies, biomarker-guided clinical trials, and multidisciplinary collaboration aimed at translating pathophysiological insights into precision reproductive medicine.
